# Editorial: COVID-19-Social Science Research During a Pandemic

**DOI:** 10.3389/fpubh.2022.923992

**Published:** 2022-05-09

**Authors:** Paul R. Ward, Paul Bissell, Samantha B. Meyer, Hailay A. Gesesew, Pande Putu Januraga, Dukjin Chang, Linda Lombi

**Affiliations:** ^1^Research Centre for Public Health Policy, Torrens University, Adelaide, SA, Australia; ^2^Deputy Vice Chancellor Research, University of Chester, Chester, United Kingdom; ^3^School of Public Health, University of Waterloo, Waterloo, ON, Canada; ^4^College of Health Sciences, Mekelle University, Mekelle, Ethiopia; ^5^Faculty of Medicine, Center for Public Health Innovation, Udayana University Denpasar, Denpasar, Indonesia; ^6^School of Sociology, Seoul National University, Seoul, South Korea; ^7^Catholic University of the Sacred Heart, Milan, Italy

**Keywords:** COVID-19, sociology, public health, social theory, coronavirus

A huge number of epidemiological, clinical and laboratory studies have been published to mitigate the coronavirus pandemic crises and the findings from these studies are helping policy makers to understand how best to manage the current and future clinical and public health responses. In addition to impacting on infection and mortality rates due to COVID-19, government responses to reducing viral spread and “flattening the curve” have meant huge impacts on social and economic life across the globe. But research is also needed to explore the social and economic impacts of COVID-19 to assist policy makers to understand the impact of current interventions and plan future policy to mitigate unintended consequences of pandemic responses. In particular, the impacts of responses which brought social disruptions such as: closing down parts of the economy and increasing unemployment, forcing some people into “social isolation”, restricting freedom of movement, closing schools/universities/workplaces, reducing democratic decision making of governments and generally disrupting the ‘social order' of the pre-COVID-19 world are less investigated.

As part of this investigation, by the 12th of May 2020, a special topic entitled “COVID-19–Social Science Research during a Pandemic” was initiated by a dedicated team of scholars as guest editors to facilitate the timely peer-review and publication of relevant manuscripts from multiple studies (1). A total of 111 manuscripts were submitted between 12 May 2020 and 1 January 2021 of which 37 manuscripts were rejected while 74 manuscripts from 298 contributing authors from all over the globe including China, Italy, US, Indonesia, and Saudi Arabia were published. Population in the studies included students, health workers, men who have sex with men (MSM), and general population.

By March 2022, the special topic achieved over 1.06 million views. In this special issue, several thematic areas were highlighted including but not limited to:

(a) Knowledge, attitude and practices to COVID-19 and its preventive measures— for example, Purnama et al. noted the continued practice of stay at home, physical distancing, and always using face masks for the public to have a supportive attitude, and Albaqawi et al. revealed good perceptions of COVID-19 knowledge and its prevention among Saudi Arabia nursing students, and positive perceptions on the government's effort in responding to the COVID-19 crisis.
(b) Policy interventions to fight COVID-19 pandemic such as pharmaceutical and non-pharmaceutical strategies— for example, Giudici and Raffinetti suggested Gini-Lorenz concentration approach to monitor COVID-19 policy interventions and Goldman's demonstrated Voluntary Cyclical Distancing as alternative approach to social distancing.
(c) Impacts of COVID-19 and its preventive measures such as increased alcohol consumption, mental illness, unintended breast cancer, human rights violations, and stigma and discrimination, and diminishing quality of life— for example, Septarini et al. reported moderate to very high psychological distress and lack of happiness during the COVID-19 pandemic among MSM in Indonesia, Lunnay et al. depicted increasing in alcohol consumption among Australian women in the emerging affluent group who experienced increased feelings or fear and anxiety during the COVID-19 pandemic, and Santos et al.'s demonstrated collision of fundamental human rights and the right to health access as a result of the preventive measures.
(d) Media and COVID-19 pandemic especially on the role of media on framing political consequences and responsibility—for example, Jo et al.'s reported media's framing on quarantine performance in South Korea bringing a positive change in people's attitudes toward the government and Thomas et al.'s added media's lack of blame of COVID-19 pandemic in Australia.
(f) Others including Trust during and post-COVID-19 pandemic such as strategies to maintain public trust, COVID-19 and HIV co-infection, poverty and death due to COVID-19, telehealth and COVID-19 pandemic, and childhood immunization and COVID-19 protection—for example, Henderson et al.'s 10 strategies of maintaining trust in public health officials, Gesesew et al. findings on intersecting nature of the COVID-19 and HIV pandemics to identify a shared research agenda using a syndemic approach, and Beric-Stojsic et al. findings on the potential protective role of BCG, MMR, and HEP-A childhood vaccines to COVID-19.

We hope that our Edited special issue provides empirical and theoretical evidence on the social impacts of the COVID-19 pandemic and food for thought for managing the social and emotional impacts of future pandemics.

## Author Contributions

All authors listed have made a substantial, direct, and intellectual contribution to the work and approved it for publication.

## Conflict of Interest

The authors declare that the research was conducted in the absence of any commercial or financial relationships that could be construed as a potential conflict of interest.

## Publisher's Note

All claims expressed in this article are solely those of the authors and do not necessarily represent those of their affiliated organizations, or those of the publisher, the editors and the reviewers. Any product that may be evaluated in this article, or claim that may be made by its manufacturer, is not guaranteed or endorsed by the publisher.
